# Quality control recommendations for RNASeq using FFPE samples based on pre-sequencing lab metrics and post-sequencing bioinformatics metrics

**DOI:** 10.1186/s12920-022-01355-0

**Published:** 2022-09-16

**Authors:** Yuanhang Liu, Aditya Bhagwate, Stacey J. Winham, Melissa T. Stephens, Brent W. Harker, Samantha J. McDonough, Melody L. Stallings-Mann, Ethan P. Heinzen, Robert A. Vierkant, Tanya L. Hoskin, Marlene H. Frost, Jodi M. Carter, Michael E. Pfrender, Laurie Littlepage, Derek C. Radisky, Julie M. Cunningham, Amy C. Degnim, Chen Wang

**Affiliations:** 1 Department of Quantitative Health Sciences, Mayo Clinic, 200 1st Street SW, Rochester, MN 55905 USA; 2 Genomics and Bioinformatics Core Facility, 019 Galvin Life Sciences Center, University of Notre Dame, Notre Dame, IN 46556 USA; 3 Department of Laboratory Medicine and Pathology, Mayo Clinic, 200 1st Street SW, Rochester, MN 55905 USA; 4 Department of Neuroscience, Mayo Clinic, 4500 San Pablo Road, Jacksonville, FL 32224 USA; 5 Department of Medical Oncology, Mayo Clinic, 200 1st Street SW, Rochester, MN 55905 USA; 6 Department of Biological Sciences, 109B Galvin Life Science Center, University of Notre Dame, Notre Dame, IN 46556 USA; 7 Department of Chemistry and Biochemistry, Harper Cancer Research Center, University of Notre Dame, Notre Dame, IN 46556 USA; 8 Department of Cancer Biology, Mayo Clinic, 4500 San Pablo Road, Jacksonville, FL 32224 USA; 9 Department of Surgery, Mayo Clinic, 200 1st Street SW, Rochester, MN 55905 USA

**Keywords:** FFPE, RNA-seq, Quality control, Breast tissue, RNA concentration, Library concentration, Decision tree, DV200, DV50

## Abstract

**Background:**

Formalin-fixed, paraffin-embedded (FFPE) tissues have many advantages for identification of risk biomarkers, including wide availability and potential for extended follow-up endpoints. However, RNA derived from archival FFPE samples has limited quality. Here we identified parameters that determine which FFPE samples have the potential for successful RNA extraction, library preparation, and generation of usable RNAseq data.

**Methods:**

We optimized library preparation protocols designed for use with FFPE samples using seven FFPE and Fresh Frozen replicate pairs, and tested optimized protocols using a study set of 130 FFPE biopsies from women with benign breast disease. Metrics from RNA extraction and preparation procedures were collected and compared with bioinformatics sequencing summary statistics. Finally, a decision tree model was built to learn the relationship between pre-sequencing lab metrics and qc pass/fail status as determined by bioinformatics metrics.

**Results:**

Samples that failed bioinformatics qc tended to have low median sample-wise correlation within the cohort (Spearman correlation < 0.75), low number of reads mapped to gene regions (< 25 million), or low number of detectable genes (11,400 # of detected genes with TPM > 4). The median RNA concentration and pre-capture library Qubit values for qc failed samples were 18.9 ng/ul and 2.08 ng/ul respectively, which were significantly lower than those of qc pass samples (40.8 ng/ul and 5.82 ng/ul). We built a decision tree model based on input RNA concentration, input library qubit values, and achieved an F score of 0.848 in predicting QC status (pass/fail) of FFPE samples.

**Conclusions:**

We provide a bioinformatics quality control recommendation for FFPE samples from breast tissue by evaluating bioinformatic and sample metrics. Our results suggest a minimum concentration of 25 ng/ul FFPE-extracted RNA for library preparation and 1.7 ng/ul pre-capture library output to achieve adequate RNA-seq data for downstream bioinformatics analysis.

**Supplementary Information:**

The online version contains supplementary material available at 10.1186/s12920-022-01355-0.

## Background

For decades, clinical biospecimens have been typically fixed in formalin then embedded in paraffin wax to make formalin-fixed paraffin-embedded (FFPE) tissue blocks for diagnosis and long-term storage. FFPE tissue archiving has many advantages, including room temperature stability, long-term storage, and suitability for subsequent immunohistochemical (IHC) analyses, which had led to use of FFPE in IHC-based biomarker investigations [1, 2]. However, FFPE processing and tissue storage are known to result in highly degraded RNAs which limits gene expression-based biomarker discovery using RNA sequencing [3–5]. Transcriptional profiling by RNA sequencing (RNA-seq) is a powerful tool for genome wide quantification of RNA expression with high sensitivity that has been routinely used in breast cancer research and clinical diagnosis [6–9]. RNA-seq involves an enrichment step to remove the abundant ribosomal RNAs by either ribosomal depletion or Poly(A) selection [10, 11]. However, Poly(A) selection protocol is less suitable for low quality RNA derived from FFPE samples [12]. During recent years, RNA library protocols tailored for FFPE samples have been developed, including the NEBNext rRNA Depletion and the TruSeq RNA Exome panel, although the relative performance of these methods with FFPE-derived RNA has not been published, and there are limited studies that provide insight for selection of FFPE samples of adequate quality [13, 14].

Our study aim is to compare two commonly used RNA library preparation protocols for FFPE samples, and to provide a recommendation on RNA input metrics, including RNA concentration and library concentration, to achieve adequate RNA-seq data for downstream bioinformatics analysis.

For the first part of the study, we evaluated two commonly used RNA library protocols for FFPE samples using seven paired FFPE and fresh frozen (FFzn) samples. All samples were prepared through both protocols and compared based on bioinformatics metrics, including alignment, SNP concordance, junction coverage and sample-wise correlation. For the second part of the study, we sequenced 130 benign breast disease (BBD) samples along with technical replicates in ten sequencing batches. Thorough bioinformatics quality control was performed to identify QC-failed samples. Finally, a decision tree model was constructed to correlate pre-sequencing metrics with QC status defined by bioinformatics metrics.

## Methods

### Study design

**Fig. 1:**
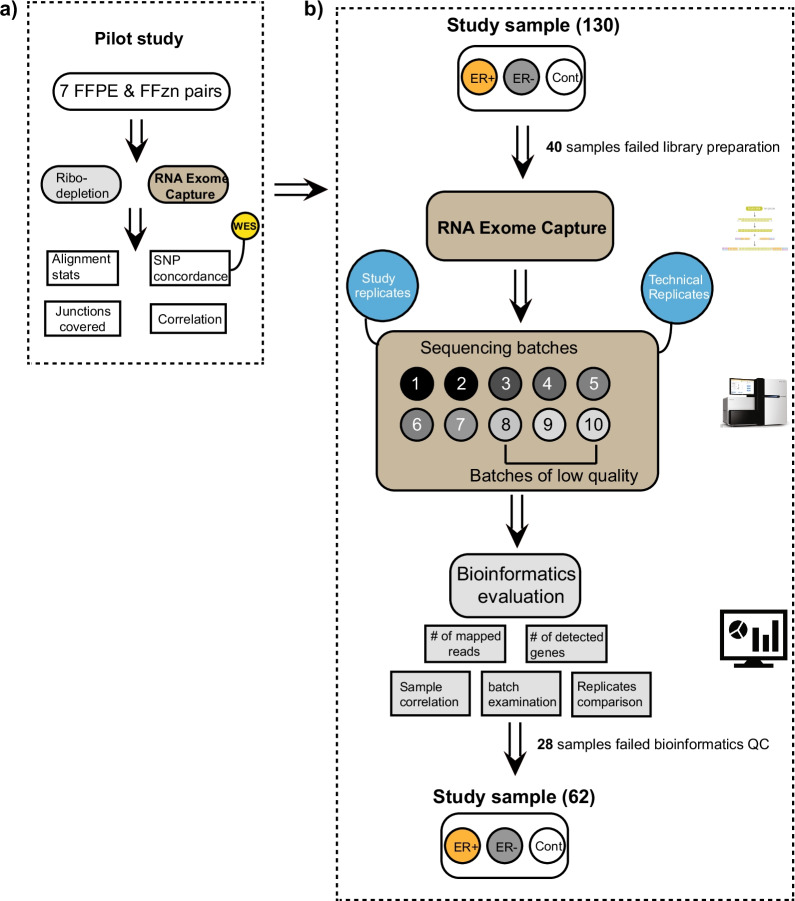
Flow-chart of library optimization and bioinformatics evaluation. **a** A pilot study consisting of FFPE and fresh frozen pairs for 7 BBD patients were submitted for sequencing to evaluate two protocols of library preparation for RNA-seq, Ribo-depletion and RNA exome capture. Several bioinformatics metrics were evaluated for the two protocols. Whole exome sequencing (WES) data was used to estimate SNP confirmation rate, and the RNA exome capture showed superior performance in all categories and was selected as the library preparation protocol to process all samples. **b** 130 study samples (*ER*+ estrogen receptor positive, *ER− *estrogen receptor negative, *Cont* control) along with 17 technical replicates and 11 study replicates were submitted for library preparation using the RNA exome capture protocol. 40 samples failed library preparation step with insufficient RNA. All remaining samples were submitted for sequencing in 10 batches. Rigorous bioinformatics evaluation was performed to identify qc failed samples based on defined bioinformatics metrics. The final dataset comprised 62 study samples

Institutional Review Board approval was obtained for research use of human samples in this project (#IRB 75–87). A pilot study was performed using FFPE and fresh frozen pairs for seven women diagnosed with benign breast disease to evaluate the performance of two library preparation protocol, Illumina’s TruSeq RNA Exome and NEBNext rRNA Depletion (Fig. 1a). To evaluate the precision of SNPs identified by the two protocols, we also performed whole exome sequencing (WES) for the three selected fresh frozen samples. The TruSeq Exome protocol exhibited better performance in bioinformatics metrics and was selected to process all study samples and technical controls in the main study. A total of 158 samples including study samples and technical controls were submitted for RNA extraction (Fig. 1b). Forty samples failed library preparation due to low RNA input quantity. The remaining samples were submitted for RNA sequencing in ten sequencing batches. Batches were designed so that samples with similar RNA quality were included in the same batch. This helped to minimize the potential sequencing bias toward high quality samples in the same batch. To examine the potential sequencing batching effect, the same two technical controls (FFPE and FFzn pair for the same subject) were included in each sequencing batch 1–7. For sequencing batch 8–10 where samples are of low RNA quality, we only included the FFPE technical control as the FFzn technical control would potentially attract more sequencing reads and bias the quantification of other low-quality study samples. Besides the two technical controls, we also included 11 study replicate samples in different sequencing batches. Thorough bioinformatics evaluations were performed to identify samples passing the qc metrics.

### RNA quantitation and quality

Total RNA concentration was determined using the Qubit 2.0 Fluorometer and RNA HS Assay (Life Technologies Corp., Carlsbad, CA). RNA integrity was assessed and recorded with DV50, DV100, and DV200 values using the RNA 6000 Nano Kit on an Agilent 2100 Bioanalyzer (Agilent Technologies, Santa Clara, CA), but was not used for sample exclusion in the library preparation. DV values are a commonly used metric that represents the proportion of RNA fragments in a sample with greater length than the numeric value (i.e., DV200 equals the percentage of RNA fragments > 200 nucleotides).

### RNA exome library preparation and sequencing

40–100 ng of experimental FFPE RNA, FFPE control RNA, or 20 ng fresh frozen control RNA was used for library preparation using the TruSeq RNA Library Prep for Enrichment and the Illumina Exome Panel-Enrichment Oligos kit (llumina, Inc., San Diego, CA) following the manufacturers protocol for FFPE RNA or high-quality total RNA respectively. As per the protocol, fragmentation of FFPE RNA was not performed. Following adaptor ligation and enrichment, the libraries were quantitated by Qubit and pooled for subsequent exome capture based on available yield. Up to a 4-plex pooling strategy was used for the exome capture, with capture groups consisting of 200 ng, 100 ng, 50 ng, 40 ng, and 30 ng of input library for each sample in the pre-capture pool. Up to 12 samples (3 pools) were batched for sequencing and included a paired FFPE control RNA and fresh frozen control RNA in each batch. Following two rounds of hybridization to the capture probes, the pools were PCR amplified and purified using AMPure XP beads. The amplified and enriched libraries were quality assessed using a combination of the Qubit dsDNA HS Assay (Invitrogen), the Bioanalyzer DNA 7500 Assay (Agilent Technologies), and KAPA Library Quantification Kit for Illumina (KAPA Biosystems, Boston, MA). The three capture pools for each batch were combined in equal molar amounts and sequenced across 3 lanes of an Illumina NextSeq 500 High Output flowcell using 75 × 2 bp paired end reads. Each flowcell generated a minimum of 700 million reads passing filter.

### rRNA depletion library preparation and sequencing

20–100 ng of FFPE RNA and paired fresh frozen RNA was used for library preparation using the NEBNext rRNA Depletion Kit (Human/Mouse/Rat) and Ultra II Directional RNA Library Prep Kit for Illumina (New England Biolabs Inc., Ipswich, MA), following the manufacturers protocol for highly degraded (RIN ≤ 2) or intact (RIN > 7) samples respectively. Fragmentation is based on RIN value of RNA input and conducted as outlined in the protocol. Fragmentation for FFPE RNA was not performed. Experimental FFPE RNA and paired fresh frozen RNA from the same patient was used if available using similar input amounts for each sample type. A total of 13 libraries were prepared, including six patient pairs. Libraries were quality assessed using a combination of the Qubit dsDNA HS Assay (Invitrogen), the Bioanalyzer DNA 7500 Assay (Agilent Technologies), and KAPA Library Quantification Kit for Illumina (KAPA Biosystems, Boston, MA). Libraries were combined in equal molar amounts and sequenced across three lanes of an Illumina NextSeq 500 High Output flowcell using 75 × 2 bp paired end reads. Each flowcell generated a minimum of 800 million reads passing filter.

### Whole exome sequencing of fresh frozen samples

Three fresh frozen samples were submitted for whole exome sequencing at Mayo Clinic molecular genomic facility. In brief, paired-end libraries were prepared with 1.0 μg of genomic DNA in accordance with the manufacturer's protocol (Agilent Technologies, Inc, Santa Clara, Calif). Whole-exon capture was performed with 750 ng of the prepped library following the protocol for the SureSelect Human All Exon v5 + UTRs 75 Mb kit (Agilent Technologies, Inc). The purified capture products were then amplified with use of SureSelect Post-Capture Indexing forward and Index polymerase chain reaction reverse primers (Agilent Technologies, Inc) for 12 cycles. Concentration and size distribution of the completed captured libraries were assessed on Qubit (Invitrogen, Waltham, Mass) and Bioanalyzer DNA 1000 chip (Agilent Technologies, Inc). Libraries were sequenced at an average coverage of about 80× in accordance with standard protocol of the cBot and HiSeq 3000/4000 PE Cluster Kit (Illumina, San Diego, Calif). The flow cells were sequenced as 150 × 2 paired end reads on the HiSeq 4000 with the HiSeq 3000/4000 sequencing kit and collection software (HCS version 3.3.52; Illumina). Base calling was performed with Real-Time Analysis version 2.7.3 (Illumina). All procedures were performed in accordance with the manufacturer's instructions.

### RNA-seq alignment and gene quantification

After sequencing procedure, raw FASTQ files were processed through Mayo’s internal MAP-RSeq pipeline [15] (Version 3.0). MAP-RSeq uses a variety of publicly available bioinformatics tools tailored by in-house developed methods. Briefly, the aligning and mapping of reads are performed using Star aligner [16] against hg38 genome build. The gene and exon counts are generated by FeatureCounts [17] using the gene definitions files from Ensembl v78. Quality control was carried out using RSeqQC [18]. Gene expression data was normalized to counts per million (CPM) and transcript per million (TPM) using Trimmed Mean of M-values (TMM) method as implemented in edgeR [19] followed by log2 transformation.

### Estimation of SNP confirmation rate and false positive rate

SNPs were identified using GATK haplotype caller [20] and further filtered by RVBoost [21]. For the pilot study, SNP confirmation rate (precision) was calculated for each mutation type (C>T, C>G, etc.) as:

1 $$SNP \,confirmation\,rate = SNP_{rna} \cap SNP_{dna} /SNP_{rna} ,$$

where *SNP*_*RNA*_ represents the SNPs identified in RNA-seq data and *SNP*_*DNA*_ represents the SNPs identified in WES data for the same set of samples. For both pilot and main study, false positive rate (FPR) between either technical or study replicate samples was calculated as:

2 $$FPR = N_{MAD > lfc} /N_{total} ,$$

where *N*_*total*_ denotes the total number of genes and *N*_*MAD*>*lfc*_ denotes the number of genes with maximum absolute difference (MAD) above a certain logarithm fold change cutoff.

### Bioinformatics QC and model building

We defined three bioinformatics metrics for QC purpose, including sample-wise median correlation of gene expression (median_cor_expr), number of genes mapped to genic regions (gene_reads), number of detectable genes with transcript per million (TPM) larger than 4 (gene_tpm4). TPM was calculated based on:

3 $$TPM = \frac{FPKM}{{\sum FPKM}} \times 10^{6} ,$$

FPKM was calculated as described earlier [22]. For each sample, we firstly calculate its Spearman rank correlation of gene expression with each of the rest of samples in the cohort. Then, ‘median_cor_expr’ for each sample is the median Spearman correlation value with the rest of samples in the cohort. After thorough bioinformatics evaluation, samples meeting any of the below criteria were flagged as QC-fail:

1. Sample-wise median gene expression correlation smaller than 0.75
2. Gene mapped reads smaller than 25 million gene mapped reads,
3. Less than 11,400 # of detected genes with TPM > 4

For each of the sequencing batch, if the technical controls/replicates failed QC, the whole batch of samples will be flagged as QC-fail as well. For our study, all technical controls/replicates have passed QC. A decision tree model was built based on CART Modeling via rpart R package to learn the relationship between pre-sequencing QC metrics, such as RNA qubit or pre-capture library qubit, and QC pass/fail status predicted by post sequencing bioinformatics metrics. Samples were split into training and testing set with a ratio of 7:3. Repeated cross validation were performed to optimal parameters (complexity parameter) during model training. Similarly, an alternative model based on logistic regression was also constructed:

4 $$\ln \left( {\frac{p\left( X \right)}{{1 - p\left( X \right)}}} \right) = \beta_{0} + \beta_{1} X$$

All models were built based on Caret R package [23] and all statistical analysis was carried out in R under R version 4.0.3.

## Results

### Evaluating FFPE library preparation kits using FFPE and fresh frozen replicates

We evaluated two RNA-seq library preparation protocols optimized for low quality, highly degraded samples (such as FFPE): Illumina’s TruSeq RNA Exome protocol and NEBNext rRNA Depletion protocol (Fig. 1a). FFPE and Fresh Frozen (FFzn) replicates for seven BBD patients were prepared using the two selected protocols and submitted for RNA sequencing as described in the Methods section. While the Depletion protocol generated more sequenced reads compared to RNA Exome, a significantly lower proportion of reads were mappable to genic or exon-exon junction regions for both FFzn and FFPE samples (Additional file 1), and captured a smaller number of canonical exon-exon junctions (Additional file 1). We also examined the two protocols in terms of their ability to accurately capture SNP genotypes. SNP confirmation rate (precision) was calculated for three FFzn samples by using SNPs identified from their corresponding DNA whole exome sequencing (WES) data (Additional file 1). The calculation was performed separately for six conventional mutation categories (C>T, C>G, C>A, T>A, T>C, and T>G.). The Depletion protocol generated many false positive calls with consistently low SNP confirmation rate across all mutation categories. For the RNA Exome protocol, the SNP confirmation rate was significantly higher across different mutation categories with C>T being highest (p value < 2.2E−16) as was previously reported [24]. Finally, we compared the two protocols in terms of their correlation with data from the TruSeq protocol with PolyA selection using five FFzn samples (Additional file 1). For the Depletion protocol, only two of five samples successfully clustered by subject ID instead of library protocol. The RNA Exome protocol showed good concordance with the TruSeq PolyA data where all samples were clustered by subject ID regardless of their library protocol. Overall, the RNA Exome protocol showed superior performance compared to the Depletion protocol in terms of these bioinformatics metrics and was selected as the library protocol to process all samples in the main study (Additional file 1, Fig. 1b).

### Sample QC based on bioinformatics metrics

**Fig. 2:**
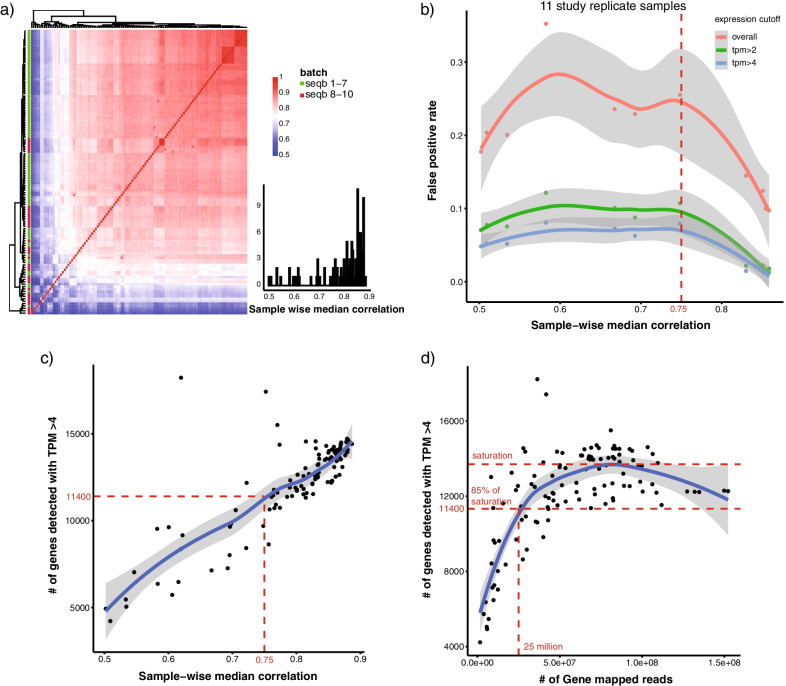
Bioinformatics QC to identify pass versus fail samples. **a** Heatmap of sample pairwise correlation of gene expression. Row color annotation bar indicate sequencing batch (seqb) 1–7 and 8–10. Right lower panel shows a histogram of the distribution of sample wise median correlation based on gene expression data. **b** Relationship between sample wise median correlation of gene expression with false positive rate using 11 study replicate samples. Samples with a sample-wise median correlation below 0.75 were classified as QC failed samples. Loess is used curve fitting and 95% confidence interval is plotted in grey bands. **c** Relationship between sample wise median correlation of gene expression with number of detectable genes with transcript per million (TPM) > 4. A cutoff of 11,400# of genes was selected to identify QC failed samples. **d** Relationship between number of gene mapped reads and total number of detected genes with transcript per million (TPM) > 4. A cutoff was selected at 80% of saturation point (20 million gene mapped reads, 10,400 # of detected genes with TPM > 4)

All study samples and technical controls were submitted for library preparation using RNA Exome protocol. Library concentration was gathered on the individual samples prior to hybridization capture and is hereafter referred to as the “pre-capture” library. 40 samples failed this step due to low pre-capture library output. The remaining samples were submitted for RNA sequencing in ten batches as detailed in the methods section (Fig. 1b). Bioinformatics quantification of gene expression was then performed, and qc metrics were collected, including sample-wise median gene expression correlation (median_cor_expr), number of gene mapped reads (gene_reads), number of detectable genes with transcript per million (TPM) larger than 4 (gene_tpm4). Median_cor_expr was calculated for each sample as its median correlation of gene expression with all other samples in the cohort, and was between 0.8 and 0.9 for most samples (Fig. 2a). Based on the 11 study sample replicates, we first evaluated the relationship between false positive rate (FPR) and median_cor_expr (Fig. 2b). Replicates with lower median_cor_expr tend to have higher FPR. For samples with extremely low median_cor_expr, FPR decreased and plateaued around 20% likely due to a reduced number of detectable genes. Similar trends were observed between FPR and median_cor_expr when applying an expression cutoff before calculating FPR. Due to the limited number of study replicates within median_cor_expr range of 0.7 and 0.8, a median_cor_expr value around the inflection point (0.75) was selected as a cutoff to identify qc failed samples. We next investigated the relationship between median_cor_expr and gene_tpm4 (Fig. 2c). Samples with higher gene_tpm4 had better median_cor_expr; a median_cor_expr of 0.75 corresponding to a value of 11,400 for gene_tpm4 was selected as the threshold. Finally, we examined the relationship between gene_reads and gene_tpm4 (Fig. 2d). Samples with increased gene_reads had higher gene_tpm4. The trend saturated with gene_tpm4 around 13,000, with a gene_tpm4 of 11,400 (roughly 85% of saturation) corresponding to 25 million gene_reads. Samples were therefore identified as QC failed when meeting any of the following criteria: 1) low median sample-wise correlation within the cohort (median_cor_expr < 0.75); 2) low number of detectable genes (gene_tpm4 < 11,400); 3) low number of reads mapped to gene regions (gene_reads < 25 million). We also evaluated the effect of sample size on the calculating of median_cor_expr (Additional file 2). Median_cor_expr are very stable with different sample sizes, and we start to achieve a good estimation even when the sample size is small (10–20 range). This confirms that our using of median correlation is robust and applicable even to small study size.

### Relationship between pre-sequencing lab metrics and post sequencing bioinformatics metrics

**Fig. 3:**
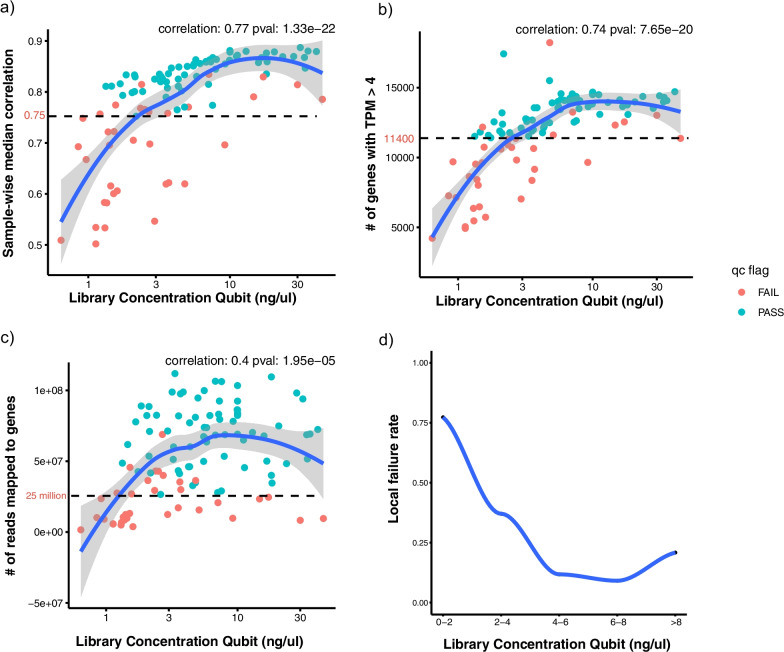
Correlation of pre-capture library concentration with bioinformatics metrics for all samples excluding FFzn controls. Scatter plot of RNA library concentration (ng/ul) with three bioinformatics metrics. Samples colored in red/green indicates qc failed/pass samples according to sample-wise median correlation, number of detected genes with TPM higher than 4, number of gene mapped reads. Spearman correlation and p value are shown on the upper right of each panel. A smooth line was fit to the data using loess and 95% confidence interval was also indicated as grey shaded area. **a** Scatter plot of library concentration with sample wise median correlation. Dashed line indicates a correlation of 0.75. **b** Scatter plot of library concentration with number of genes with transcript per million (TPM) higher than 4. Dashed line indicates 11,400 genes. **c** Scatter plot of library concentration with total number of gene mapped reads. Dashed line indicates 25 million gene mapped reads. **d** Scatter plot of library concentration with estimated local/regional failure rate calculated based on bioinformatics metrics within each window of library concentration

We next examined the relationship between pre-sequencing RNA metrics and post sequencing bioinformatics metrics. Among those pre-sequencing lab metrics, pre-capture library qubit values showed high correlation with bioinformatics metrics, including median_cor_expr, gene_tpm4 and gene_reads (Additional file 3). Samples failing bioinformatics qc had significantly lower library qubit values compared to qc-passed samples (p value = 2.8E−6). Figure 3 shows the detailed relationship of library qubit values with bioinformatics metrics, including median_cor_expr, gene_tpm4 and gene_reads, which were all positively correlated with library qubit (Fig. 3a–c). Local failure rate was calculated based on these bioinformatics metrics under different library qubit values. As shown in Fig. 3d, local failure rate decreased with increasing library qubit values and saturated at 20% with library qubit value around 2–4 ng/ul. We observed a similar trend with input RNA qubit (Additional file 4). Local failure rate decreased with increasing RNA qubit values and saturated at 25% with an RNA qubit value ~ 20 to 30 ng/ul. The recommended quantities of starting FFPE material according to the vendor corresponds to a range of DV200 values, with the lowest recommended quality at DV200 of 30–50%, and lowest corresponding input of 4.7 ng/ul. Recommendations for input RNA using DV50 or DV100 values has not been evaluated by the vendor.

### Prediction of QC failed samples based on pre-sequencing metrics

**Fig. 4:**
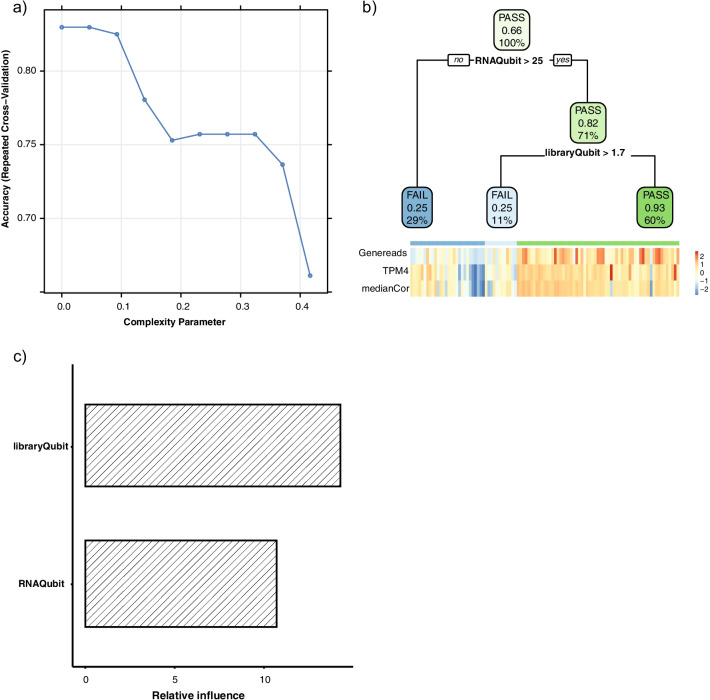
A decision tree model to predict QC pass/fail based on pre-sequencing lab metrics. QC pass and fail refer to sample status defined by bioinformatics metrics; QC failed samples were those excluded from the final dataset. **a** Parameter tuning based on repeated cross validation using grid search with 10 choices of complexity parameter. Complexity parameter with the highest cross-validation accuracy was used to build the final model. **b** Decision tree diagram with branches indicating specific cutoffs based on pre-sequencing metrics that were predictive of the qc pass/fail status. Samples with RNA qubit higher than 25 ng/ul and pre-capture library qubit higher than 1.7 ng/ul shows the best RNA-seq data quality. There are three values in each box/node. The upper value (PASS/FAIL) in each box indicates the predicted qc status based on pre-sequencing lab metrics at each branch of decision tree. The middle number in each box indicates the ratio of qc-pass samples as defined by bioinformatics metrics. The bottom number in each box indicates the percentage of total number of samples within each box. The lower panel indicates a heatmap of the three metrics (number of gene mapped reads, number of detected genes with TPM higher than 4, sample-wise median correlation) that were used to define QC status. The upper annotation bar of the heatmap indicates the three leaf nodes predicted by the decision tree. **c** Relative contribution/influence of the pre-sequencing lab metrics in building the final model

We next built a decision tree model to learn the relationship between pre-sequencing lab metrics and qc pass/fail status as determined by bioinformatics metrics. Samples were split into training and testing sets with a ratio of 7:3. Repeated cross validation was used to determine the optimal ‘complexity’ parameter used to build the training model (Fig. 4a). We found that pre-capture library concentration had higher feature importance compared to RNA concentration (Fig. 4c). Finally, we evaluated the performance of the training model by applying it to the testing set and were able to achieve an F score of 0.848. As shown in Fig. 4b, we grouped the samples into three categories based on RNA and pre-capture library concentrations: 1. Low/marginal quality (RNA qubit < 25 ng/ul); 2. Intermediate quality (RNA qubit ≥ 25 ng/ul and library qubit < 1.7 ng/ul); 3. Good quality (RNA qubit ≥ 25 ng/ul and library qubit ≥ 1.7 ng/ul). The decision tree-based model was chosen due to its high interpretability. Logistic regression analysis on the model achieved a similar performance in terms of F score (0.844).

### False positives evaluation based on FFPE and fresh frozen replicates

We also evaluated the reproducibility across sequencing batches using FFPE and FFzn replicates (Additional file 5). As expected, FFPE replicates had higher overall FPR compared to FFzn replicates (18.4% vs. 12.7%). By applying an expression cutoff of tpm > 4, FPR for both FFPE and FFzn replicates decreased significantly (1.35e−2 vs. 4.19e−3). We further investigated the relationship between MAD (Max Absolute Difference) and gene-level features including gene length and GC content for FFPE replicates. As shown in Additional file 5, shorter genes were more variable and had larger MAD compared to longer genes. GC content had a moderate positive correlation with MAD indicating that genes with high GC content were more likely to be influenced by FFPE procedure. In summary, FFPE replicates showed similar reproducibility as FFzn replicates across sequencing batches. Genes with short length or high GC content are more likely to be influenced by FFPE procedure.

## Discussion

In this study, we evaluated two commonly used RNA library protocols for FFPE samples: RNA exome capture and rRNA-depletion, using seven paired FFPE-FFzn samples. Samples processed using the RNA exome capture protocol showed a higher percentage of gene mapped reads, captured a higher number of canonical junctions, generated better SNP concordance rate and demonstrated better concordance with TruSeq PolyA data. Next, we sought to identify pre-sequencing metrics that could be used to predict sample pass/fail status based on post-sequencing bioinformatics metrics. All study samples along with replicate samples were processed using the RNA exome protocol. Three bioinformatics metrics were determined to identify qc-failed samples, including sample-wise median correlation (median_cor_expr), number of gene mapped reads (gene_reads), number of detectable genes with transcript per million (TPM) larger than 4 (gene_tpm4). Finally, a decision tree-based model was built to examine the relationship between pre-sequencing lab metrics and qc-status as defined by post-sequencing bioinformatics metrics. Based on the model, we recommend a minimum of 25 ng/ul for RNA concentration and 1.7 ng/ul for pre-capture library concentration for FFPE samples to generate good quality RNA-seq data for bioinformatics analysis. We also demonstrated that FFPE replicates have similar reproducibility compared to FFzn replicates across sequencing batches. However, genes with short length or high GC content are more likely to be influenced by the FFPE procedure.

Clinical biospecimens are typically stored as FFPE blocks, representing an invaluable source of material for biomedical research. FFPE blocks enable prolonged storage of clinical samples, preserving both tissue morphology and nucleic acids information. However, FFPE processing and tissue storage have been shown to affect RNA quality, thus limiting gene expression quantification by technologies like RNA sequencing. Our study provides a guideline for future research that utilizes FFPE samples for RNA-seq. By following these recommendations, sequencing samples with RNA and library input higher than our recommended values will not only help yield a better success rate for RNA sequencing, but also help to prevent unnecessary cost for sequencing.

There are several limitations for our study. Firstly, we benchmarked two commonly used library preparation protocols for FFPE samples using bioinformatics metrics, including SNP confirmation rate. SNP confirmation rate (precision) was calculated as the percentage of true SNPs (called by WES data) within the SNPs identified by RNA-seq for the same sample. This does not consider RNA specific mutations introduced by events like RNA editing. However, RNA editing events are considered very rare and the expected SNP confirmation rate should be very close to our calculation in Additional file 1 [25]. Allele specific gene expression could lead to discordance between SNP calls generated from RNA-seq and WES, e.g., RNA-seq might fail to capture mutations where the non-mutant allele is expressed [26]. This is also the reason that we are focusing on precision rather than sensitivity of SNPs called by RNA-seq and WES. Secondly, when performing bioinformatics QC using replicate samples, due to the limited number of replicate samples with median_cor_expr around 0.7 and 0.8 range, we arbitrarily selected a cutoff value (0.75) around the inflection point of the loess-fitted curve between median_cor_expr and FPR. This criterion will potentially affect our definition of qc pass/fail as determined by those bioinformatics metrics. To provide the user with more flexibility in selecting cutoffs for those bioinformatics metrics, we have provided a documentation that enables the end-user to define customized cutoffs based on their preference of stringency: https://github.com/Liuy12/FFPEinput. Thirdly, the concentration of RNA in the original samples is highly dependent on the amount of input tissue, original handling and storage of the sample, the extraction method used, and perhaps most importantly, the elution volume used following extraction and purification. It is difficult to compare these amounts across samples or studies unless all these factors are controlled. The library concentrations are more comparable since they are based on a consistent total RNA amount going into the library prep. Finally, bioinformatics metrics in this study were derived from breast tissue and might not be readily applicable to other tissue types, but our recommendations for study design and bioinformatics QC procedure can be tailored for other studies involving different tissue types.

Other than RNA and library input metrics, we also investigated other pre-sequencing lab metrics including DV50, DV100, DV200 values. The recommended quantities of starting FFPE material according to the vendor corresponds to a range of DV200 values, with the lowest recommended quality at DV200 of 30–50%. Recommendations for input using DV50 or DV100 values has not been evaluated by the vendor. Due to the RNA input limit, we were only able to quantify around 70% of all study samples for DV metrics. Based on those limited data, we observed that DV50 is highly correlated with DV100 values. Both DV50 and DV100 have moderate correlation with DV200, a conventional metric for measuring RNA quality (Additional file 6). DV50 value is identified as the top predictive feature for sample failure using a recursive feature elimination algorithm. Including DV50 in building the decision tree model showed similar performance compared to using RNA/library input metrics alone (Additional file 7). We suspect that this could be due to the decreased sample size with available DV values. According to the model, samples with DV50 values bigger than 82 are more likely to generate successful RNAseq data. We have included a detailed table containing all sample-related metrics (Additional file 8).

## Conclusions

We benchmarked two commonly used library preparation protocols for FFPE samples. The TruSeq RNA exome capture protocol showed a superior performance. We also provide a common bioinformatics quality control recommendation for FFPE samples. Based on our defined bioinformatics criteria, we recommend a minimum of 25 ng/ul for RNA concentration and 1.7 ng/ul for pre-capture library concentration for FFPE samples to achieve adequate RNA-seq data for downstream bioinformatics analysis.

## Supplementary Information

**Additional file 1.** Bioinformatics evaluation of library preparation protocols using FFPE and FFzn pairs.

**Additional file 2.** Evaluation of sample size on the calculation of sample wise median correlation.

**Additional file 3.** Pair-wise scatter plot among pre-sequencing lab metrics and post sequencing bioinformatics metrics.

**Additional file 4.** Correlation of pre-capture library concentration with bioinformatics metrics for all samples excluding FFzn controls.

**Additional file 5.** Evaluation of false positives based on FFPE and FFzn replicates.

**Additional file 6.** Pair-wise scatter plot among pre-sequencing lab metrics and DV50/100/200 metrics.

**Additional file 7.** A decision tree model to predict QC pass/fail based on DV50.

**Additional file 8.** All sample related metrics including pre-sequencing lab metrics and post sequencing bioinformatics metrics.

## Data Availability

Documentation is provided to enable the end-user to define customized cutoffs based on their preference of stringency: https://github.com/Liuy12/FFPEinput. Raw sequencing data are available from the corresponding author on reasonable request.

## Abbreviations

FFPE: Formalin-fixed paraffin-embedded
IHC: Immunohistochemical
RNA-seq: RNA sequencing
FFzn: Fresh frozen
BBD: Benign breast disease
ER+: Estrogen receptor positive
ER−: Estrogen receptor negative
Cont: Control
WES: Whole exome sequencing
median_cor_expr: Sample-wise median gene expression correlation
gene_reads: Number of gene mapped reads
CPM: Counts per million
TPM: Transcript per million
gene_tpm4: Number of detectable genes with TPM larger than 4
FPR: False positive rate
DV200: The percentage of RNA fragments > 200 nt
DV100: The percentage of RNA fragments > 100 nt
DV50: The percentage of RNA fragments > 50 nt
MAD: Max absolute difference
seqb: Sequencing batch
TMM: Trimmed mean of M-values
