# Supplementary material for: Quality control recommendations for RNASeq using FFPE samples based on pre-sequencing lab metrics and post-sequencing bioinformatics metrics
Source: BMC Med Genomics. 2022 Sep 16;15:195. doi: 10.1186/s12920-022-01355-0 (PMC9479231; doi:10.1186/s12920-022-01355-0)
Supplement: Supplementary file 1 — Additional file 1. Bioinformatics evaluation of library preparation protocols using FFPE and FFzn pairs. [file 12920_2022_1355_MOESM1_ESM.pdf]

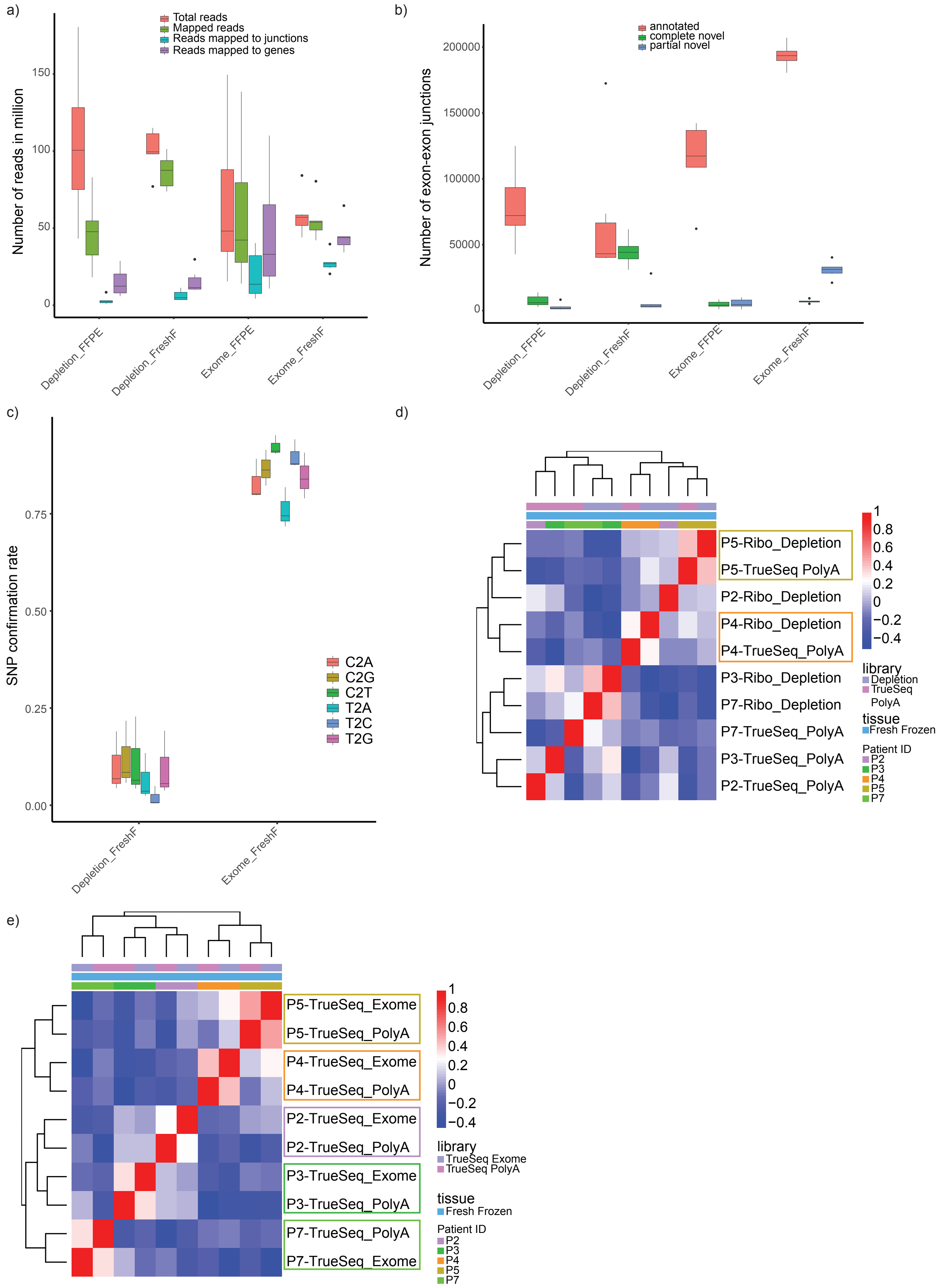
a)
Total reads
Mapped reads
Reads mapped to junctions
Reads mapped to genes
Number of reads in million
150
100
50
0
Depletion_FFPE
Depletion_FreshF
Exome_FFPE
Exome_FreshF
b)
annotated
complete novel
partial novel
Number of exon-exon junctions
200000
150000
100000
50000
0
Depletion_FFPE
Depletion_FreshF
Exome_FFPE
Exome_FreshF
c)
SNP confirmation rate
0.75
0.50
0.25
0.00
C2A
C2G
C2T
T2A
T2C
T2G
Depletion_FreshF
Exome_FreshF
d)
P5-Ribo_Depletion
P5-TrueSeq PolyA
P2-Ribo_Depletion
P4-Ribo_Depletion
P4-TrueSeq_PolyA
P3-Ribo_Depletion
P7-Ribo_Depletion
P7-TrueSeq_PolyA
P3-TrueSeq_PolyA
P2-TrueSeq_PolyA
1
0.8
0.6
0.4
0.2
0
−0.2
−0.4
library
Depletion
TrueSeq PolyA
tissue
Fresh Frozen
Patient ID
P2
P3
P4
P5
P7
e)
P5-TrueSeq_Exome
P5-TrueSeq_PolyA
P4-TrueSeq_Exome
P4-TrueSeq_PolyA
P2-TrueSeq_Exome
P2-TrueSeq_PolyA
P3-TrueSeq_Exome
P3-TrueSeq_PolyA
P7-TrueSeq_PolyA
P7-TrueSeq_Exome
1
0.8
0.6
0.4
0.2
0
−0.2
−0.4
library
TrueSeq Exome
TrueSeq PolyA
tissue
Fresh Frozen
Patient ID
P2
P3
P4
P5
P7

Additional file 1
Depletion and RNA exome capture protocols were examined using FFPE and FFzn pairs for seven BBD patients:
a). Boxplot of alignment statistics, showing total number of reads (orange), number of mapped reads (green), number of reads mapped to exon-exon junctions (blue) and number of reads mapped to genes (purple).
b). Boxplot of number of captured canonical or novel exon-exon junctions, annotated (orange), completely novel (blue), partially novel (purple).
c). SNP confirmation rate based on DNA exome-seq samples, showing the six nucleotide substitution categories for FFzn samples with each sequencing protocol
d). Heatmap of gene expression correlation matrix between 5 BBD FFzn samples processed with either Ribo-Depletion protocol or TrueSeq protocol with polyA selection. The top column annotation color bar indicates library preparation protocol. The middle column annotation color bar indicates tissue type. The lower column annotation color bar indicates patient ID. The rectangle boxes indicate samples clustered by patient ID rather than technology type.
e). Heatmap of gene expression correlation matrix between 5 BBD FFzn samples processed with either RNA exome capture protocol (no polyA selection) or TrueSeq protocol with polyA selection. The top column annotation color bar indicates library preparation protocol. The middle column annotation color bar indicates tissue type. The lower column annotation color bar indicates patient ID. The rectangle boxes indicate samples clustered by patient ID rather than technology type.
