# Supplementary material for: Quality control recommendations for RNASeq using FFPE samples based on pre-sequencing lab metrics and post-sequencing bioinformatics metrics
Source: BMC Med Genomics. 2022 Sep 16;15:195. doi: 10.1186/s12920-022-01355-0 (PMC9479231; doi:10.1186/s12920-022-01355-0)
Supplement: Supplementary file 2 — Additional file 2. Evaluation of sample size on the calculation of sample wise median correlation. [file 12920_2022_1355_MOESM2_ESM.pdf]

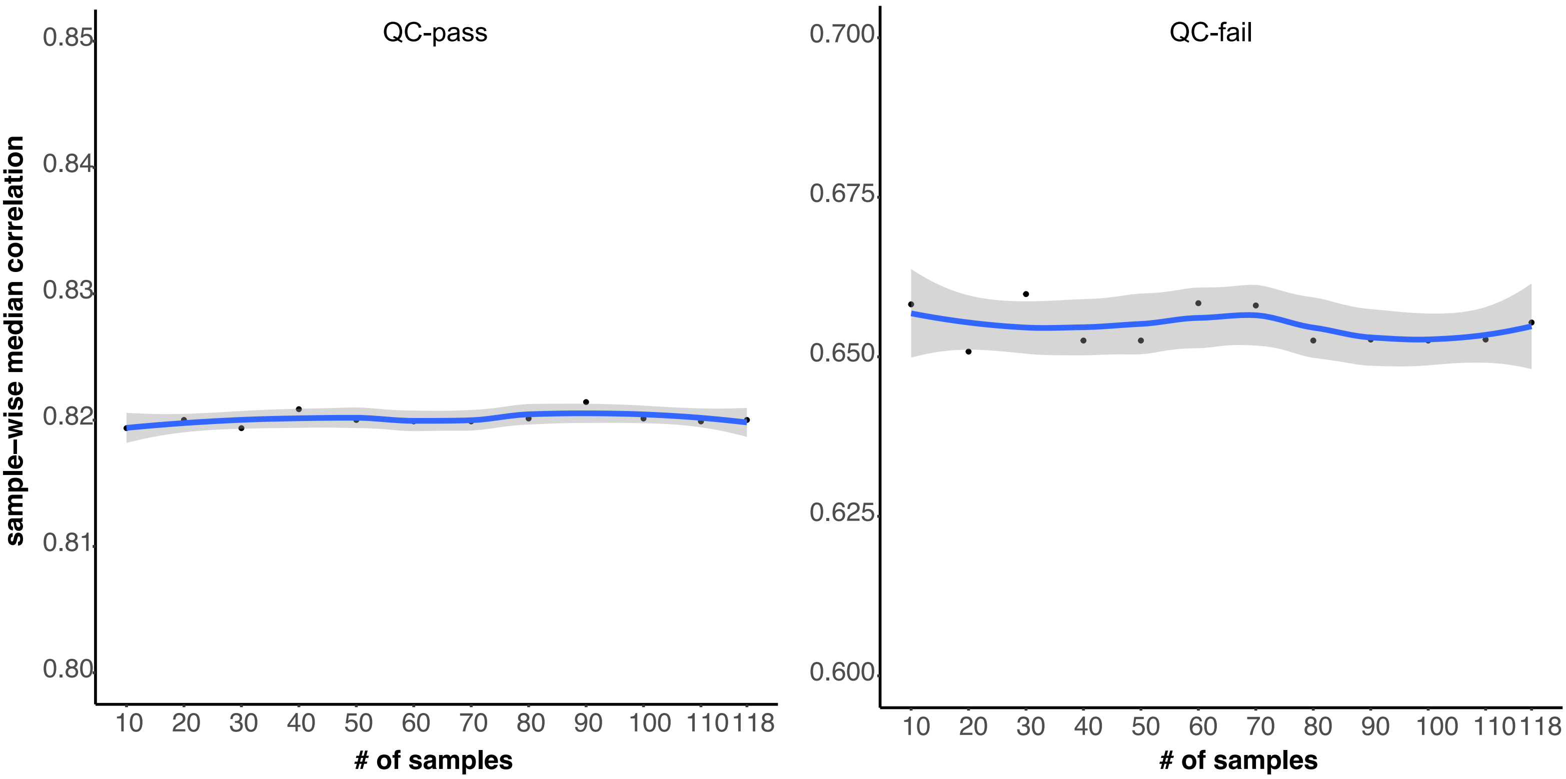

Additional file 2

Evaluation of sample size on the calculation of sample wise median correlation

X axis indicates # of samples based on sub-sampling. Y axis shows re-calculated sample-wise median correlation for each subset. Left panel shows “median_cor_expr” for one selected QC-pass sample. Right panel shows “median_cor_expr” for one selected QC-fail sample
