# Supplementary material for: Quality control recommendations for RNASeq using FFPE samples based on pre-sequencing lab metrics and post-sequencing bioinformatics metrics
Source: BMC Med Genomics. 2022 Sep 16;15:195. doi: 10.1186/s12920-022-01355-0 (PMC9479231; doi:10.1186/s12920-022-01355-0)
Supplement: Supplementary file 3 — Additional file 3. Pair-wise scatter plot among pre-sequencing lab metrics and post sequencing bioinformatics metrics. [file 12920_2022_1355_MOESM3_ESM.pdf]

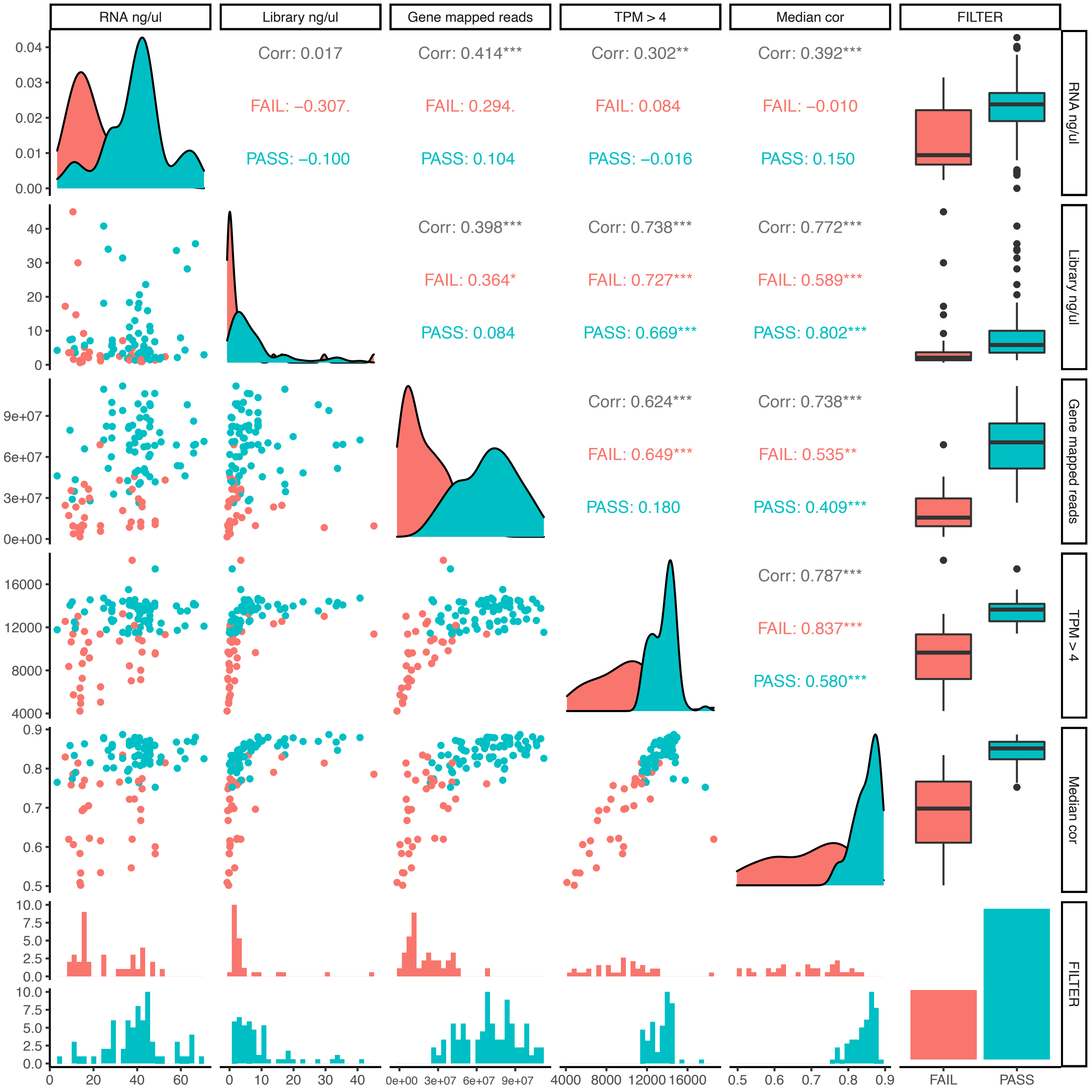

Additional file 3
Pair-wise scatter plot among pre-sequencing lab metrics and post sequencing bioinformatics metrics
Pre-sequencing lab metrics include RNA concentration and library concentration (ng/ul). Post sequencing bioinformatics metrics include number of gene mapped reads, sample wise median correlation, number of detected genes with TPM>4. Scatter plots within the left triangle illustrate the relationship between two metrics colored by bioinformatics qc status (red for FAIL, green for PASS). The diagonal histograms indicate distribution of a certain metric separated by QC status. The upper triangle shows the spearman correlation between two metrics. Significant correlations were marked with *. Correlations were also calculated separately for qc-pass and qc-fail groups. The rightmost column shows boxplots of a certain metric grouped by qc status.
