# Supplementary material for: Quality control recommendations for RNASeq using FFPE samples based on pre-sequencing lab metrics and post-sequencing bioinformatics metrics
Source: BMC Med Genomics. 2022 Sep 16;15:195. doi: 10.1186/s12920-022-01355-0 (PMC9479231; doi:10.1186/s12920-022-01355-0)
Supplement: Supplementary file 4 — Additional file 4. Correlation of pre-capture library concentration with bioinformatics metrics for all samples excluding FFzn controls. [file 12920_2022_1355_MOESM4_ESM.pdf]

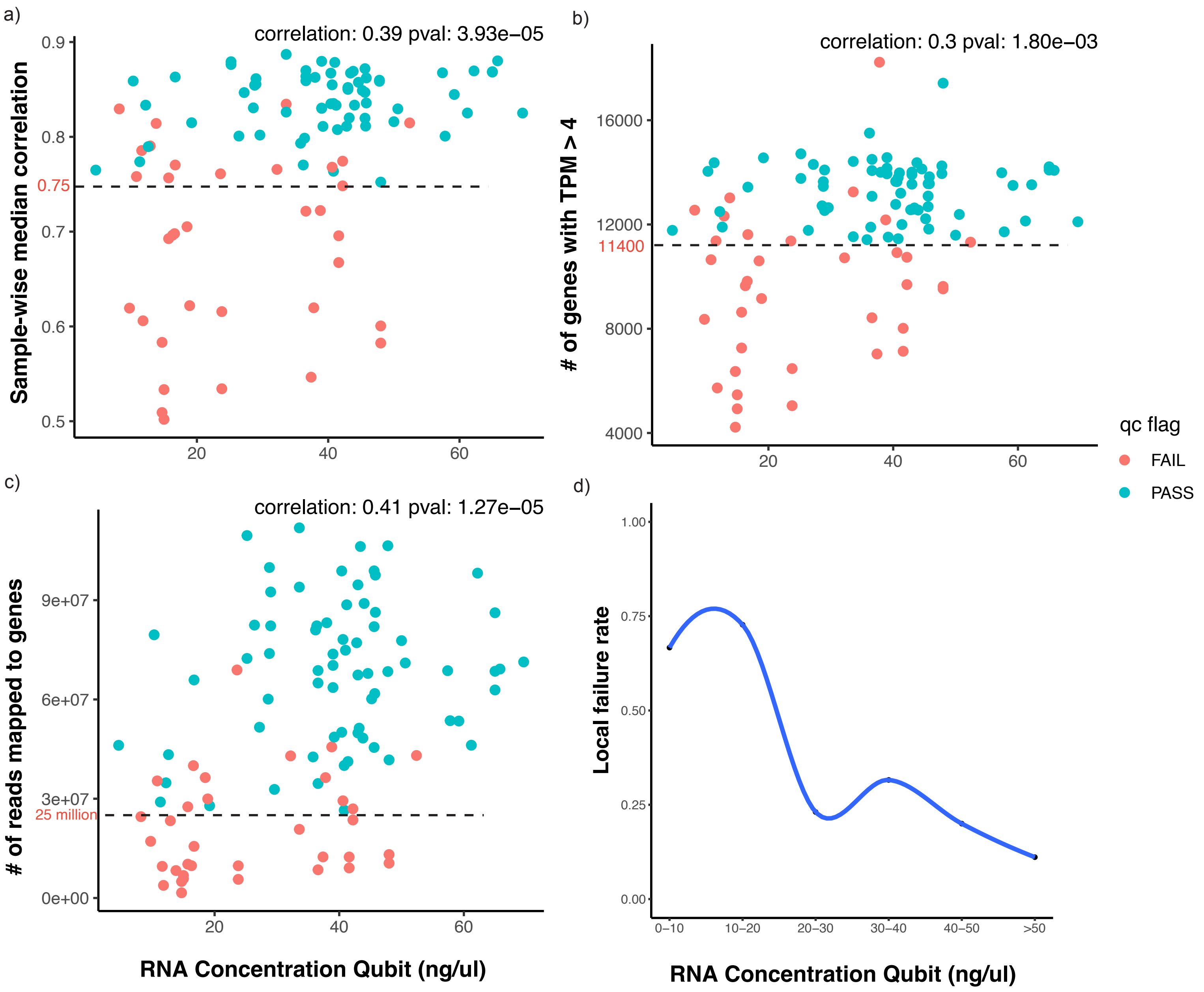

Additional file 4

Scatter plot of RNA concentration qubit (ng/ul) with three bioinformatics metrics. Samples colored in red/green indicates qc failed/pass samples according to sample-wise median correlation, number of detected genes with TPM higher than 4, number of gene mapped reads. Spearman correlation and p value are shown on the upper right of each panel. A smooth line was fit to the data using loess and 95% confidence interval was also indicated as grey shaded area.

a). Scatter plot of RNA concentration with sample wise median correlation. Dashed line indicates a correlation of 0.75

b). Scatter plot of RNA concentration with number of genes with transcript per million (TPM) higher than 4. Dashed line indicates 11,400 genes

c). Scatter plot of RNA concentration with total number of gene mapped reads. Dashed line indicates 25 million gene mapped reads

d). Scatter plot of RNA concentration with estimated local/regional failure rate calculated based on bioinformatics metrics within each window of library concentration.
