# Supplementary material for: Quality control recommendations for RNASeq using FFPE samples based on pre-sequencing lab metrics and post-sequencing bioinformatics metrics
Source: BMC Med Genomics. 2022 Sep 16;15:195. doi: 10.1186/s12920-022-01355-0 (PMC9479231; doi:10.1186/s12920-022-01355-0)
Supplement: Supplementary file 5 — Additional file 5. Evaluation of false positives based on FFPE and FFzn replicates. [file 12920_2022_1355_MOESM5_ESM.pdf]

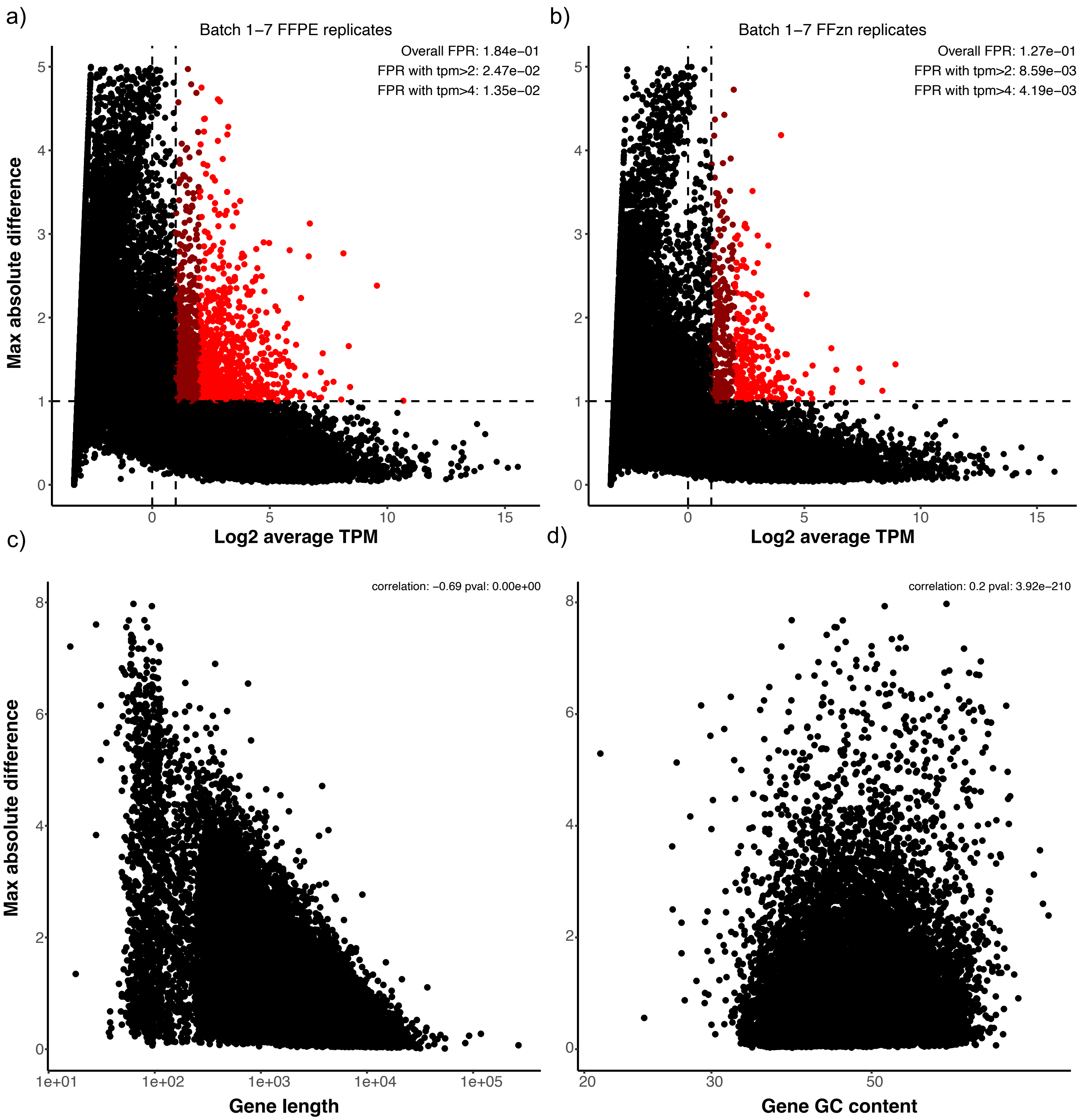

Additional file 5

a). Scatter plot of log2 average expression with max absolute differences among FFPE replicates in sequencing batch 1 and batch 7. Vertical dashed lines indicate log2 average expression of 0 and 1. Horizontal dashed line indicates max absolute differences of 1 (2-fold change). False positives are shown in red. The false positive rate at different cutoffs is shown in the top right-hand corner.

b). Scatter plot of log2 average expression with max absolute differences among FFzn replicates in sequencing batch 1 to batch 7.

c). Scatter plot of gene length with max absolute differences among FFPE replicates in sequencing batch 1 to batch 7

d). Scatter plot of gene GC content with max absolute differences among FFPE replicates in sequencing batch 1 to batch 7
