# Supplementary material for: Quality control recommendations for RNASeq using FFPE samples based on pre-sequencing lab metrics and post-sequencing bioinformatics metrics
Source: BMC Med Genomics. 2022 Sep 16;15:195. doi: 10.1186/s12920-022-01355-0 (PMC9479231; doi:10.1186/s12920-022-01355-0)
Supplement: Supplementary file 6 — Additional file 6. Pair-wise scatter plot among pre-sequencing lab metrics and DV50/100/200 metrics. [file 12920_2022_1355_MOESM6_ESM.pdf]

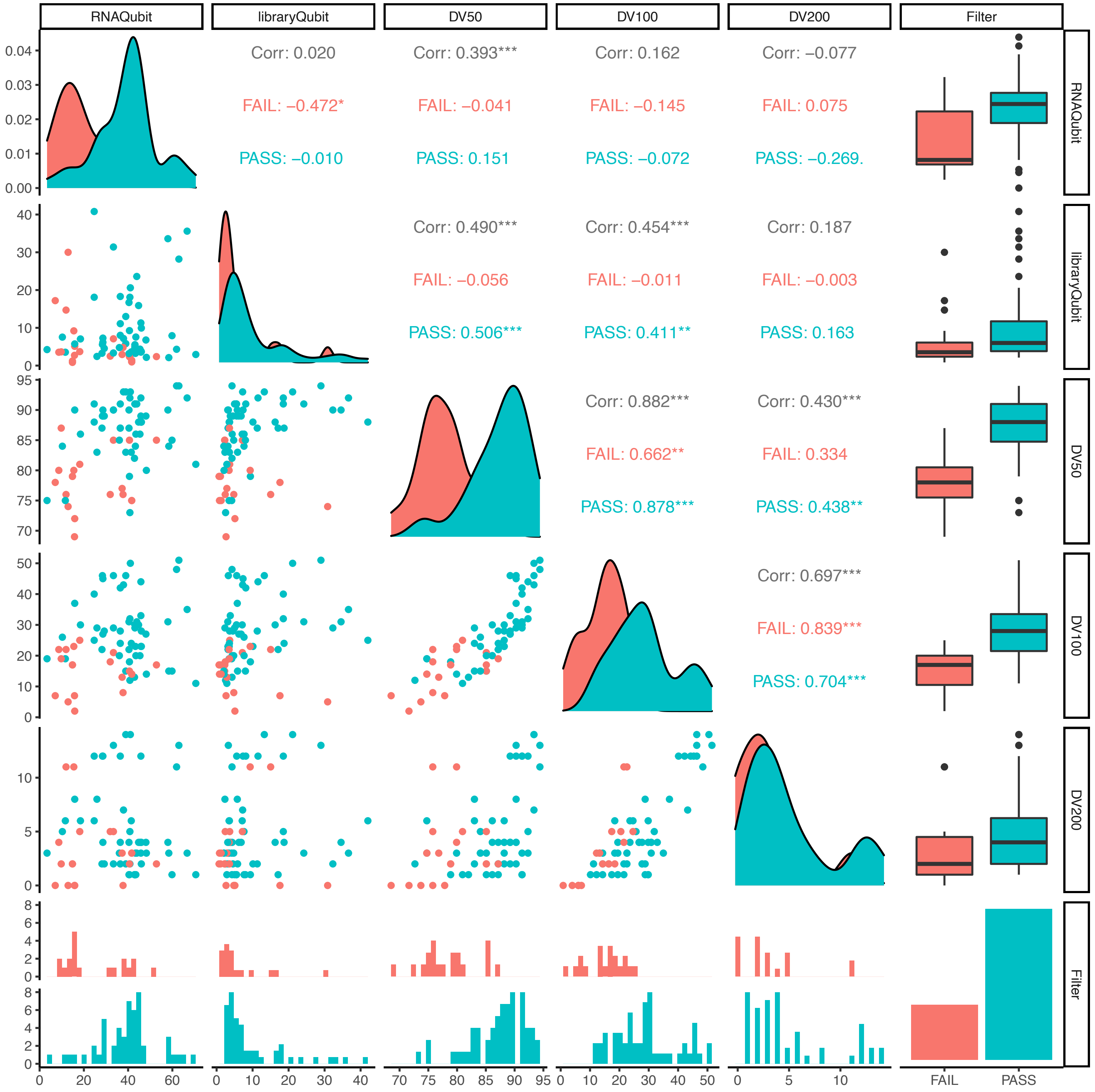

Additional file 6

Pair-wise scatter plot among pre-sequencing lab metrics and DV metrics

Pre-sequencing lab metrics include RNA concentration and library concentration (ng/ul). DV metrics include DV50, DV100, DV200. Scatter plots within the left triangle illustrate the relationship between two metrics colored by bioinformatics qc status (red for FAIL, green for PASS). The diagonal histograms indicate distribution of a certain metric separated by QC status. The upper triangle shows the spearman correlation between two metrics. Significant correlations were marked with *. Correlations were also calculated separately for qc-pass and qc-fail groups. The rightmost column shows boxplots of a certain metric grouped by qc status
