# Supplementary material for: Quality control recommendations for RNASeq using FFPE samples based on pre-sequencing lab metrics and post-sequencing bioinformatics metrics
Source: BMC Med Genomics. 2022 Sep 16;15:195. doi: 10.1186/s12920-022-01355-0 (PMC9479231; doi:10.1186/s12920-022-01355-0)
Supplement: Supplementary file 7 — Additional file 7. A decision tree model to predict QC pass/fail based on DV50. [file 12920_2022_1355_MOESM7_ESM.pdf]

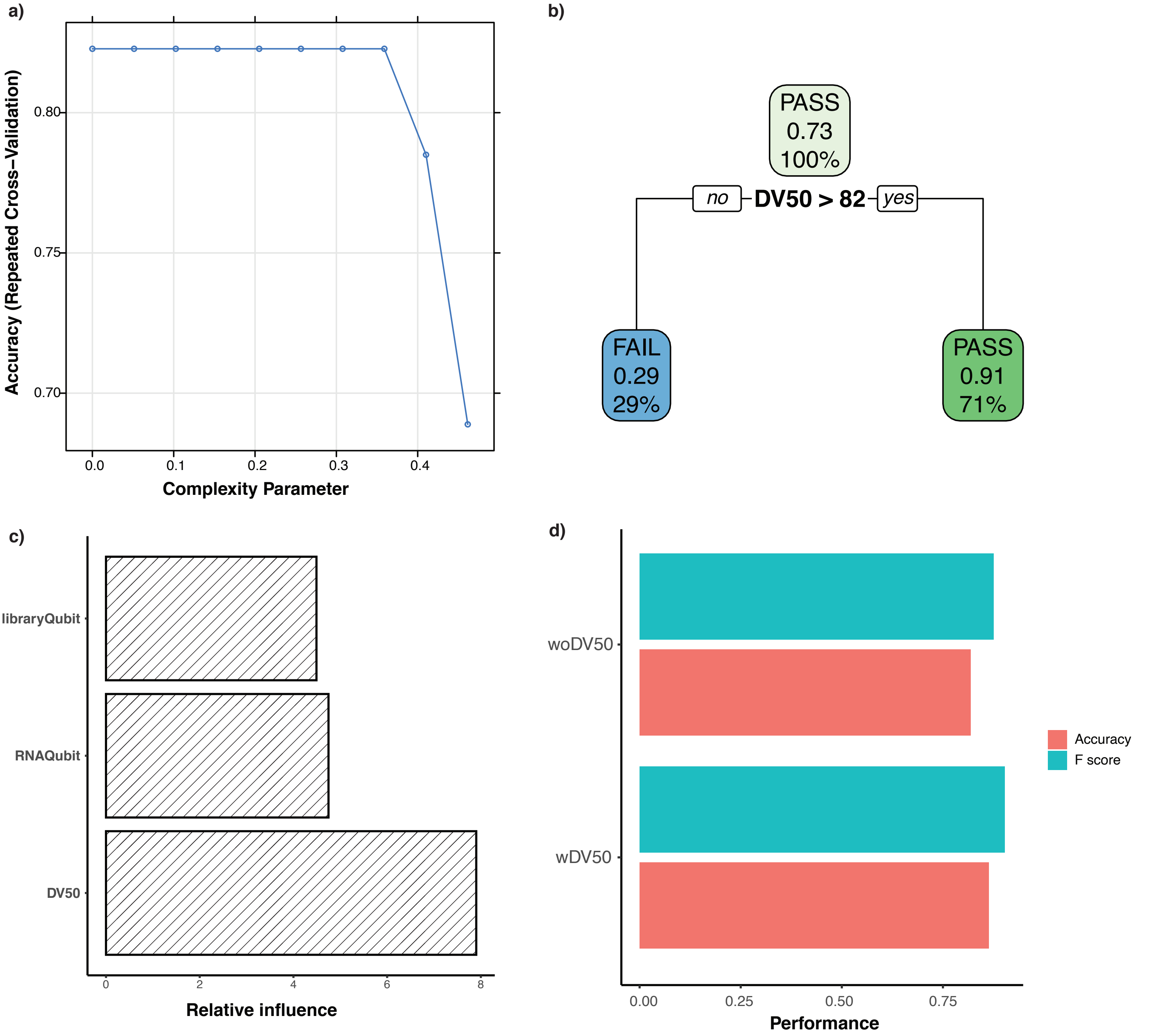

Additional file 7

A decision tree model to predict QC pass/fail based on DV50

QC pass and fail refer to sample status defined by bioinformatics metrics; QC failed samples were those excluded from the final dataset.

a). Parameter tuning based on repeated cross validation using grid search with 10 choices of complexity parameter. Complexity parameter with the highest cross-validation accuracy was used to build the final model

b). Decision tree diagram with branches indicating specific cutoffs based on DV50 that was predictive of the qc pass/fail status. Samples with DV50 > 82 shows the best RNA-seq data quality. There are three values in each box/node. The upper value (PASS/FAIL) in each box indicates the predicted qc status based on DV50 at each branch of decision tree. The middle number in each box indicates the ratio of qc-pass samples as defined by bioinformatics metrics. The bottom number in each box indicates the percentage of total number of samples within each box.

c). Relative contribution of the pre-sequencing lab metrics, including library Qubit, RNA Qubit and DV50 in building the model

d). Performance comparison (Accuracy and F score) between models with or without DV50 metric.
